# A122 OUTCOMES OF COLD SNARE POLYPECTOMY FOR POLYPS GREATER THAN 20 MM: DATA FROM AN ACADEMIC CENTER

**DOI:** 10.1093/jcag/gwab049.121

**Published:** 2022-02-21

**Authors:** J Johar, A R Kohansal, D Farina, S E Gruchy, G Williams, S Patel

**Affiliations:** 1 Medicine, Dalhousie University, Halifax, NS, Canada; 2 Dalhousie University, Halifax, NS, Canada

## Abstract

**Background:**

Cold snare polypectomy (CSP) is widely utilized for resection of small polyps (less than 10mm), due to the technique’s favourable safety profile. However, Hot Snare Endoscopic Mucosal Resection (HSEMR) remains the standard of care for large polyps greater than or equal to 20mm. HSEMR carries an increased risk of complications. These include delayed bleeding, perforation, and post-polypectomy syndrome, along with residual or recurrent adenomatous (RRA).

More recently, Cold Snare Endoscopic Mucosal Resection (CSEMR) technique has been used selectively for polyps greater than 20mm. Published studies have demonstrated a significantly lower adverse event rate of complications as compared with HSEMR. RRA utilizing CSP remains a concern, with a recent publication reporting a recurrence of 34.8% on follow-up endoscopy. We present the experience at a single Canadian academic centre with patients undergoing CSEMR for polyps greater than 20mm.

**Aims:**

To demonstrate the efficacy and safety of cold snare endoscopic mucosal resection CSEMR of large (greater than or equal to 20mm) polyps at a single Canadian academic centre.

**Methods:**

We retrospectively reviewed colonoscopies performed by endoscopists employing this technique at our centre from January 2020 to August 2021. 15 total cases were identified for patients with polyps greater than or equal to 20 mm, removed by CSEMR technique.

**Results:**

Patient age ranged from 41 to 76, with a median patient age was 66. There were 10 males and 5 females. There were no adverse events intraoperatively. 15/15 (100%) polyps were successfully resected using CSEMR technique. Polyp size ranged from 2 cm to 7 cm, and there was a median polyp size of 3 cm. 14/15 (93%) polyps were proximal to the splenic flexure, with 1/15 (7%) polyp in the rectum. 6/15 (40%) patients received snare tip soft coagulation to the edges of the polypectomy site. Polyp histology included 7 tubular adenomas, 3 tubulovillous adenomas, 4 sessile serrated polyps, and one hyperplastic polyp. Two patients were on aspirin 81 mg daily, and there were zero patients on any other antiplatelets or anticoagulation. 1/15 patient presented with late bleeding requiring emergent colonoscopy. There were no immediate or delayed perforations or other serious adverse events recorded.

Approximately 50% of patients have had follow-up colonoscopy within 3–6 months of their initial procedure. Follow-up ranged from 12 to 252 days, with a median time to follow up of 171 days. 3/7 (43%) patients had histologic evidence of recurrence at follow-up colonoscopy, all of which were successfully treated endoscopically.

**Conclusions:**

Selective CSEMR is a safe and effective technique, with a low risk of complications. There may be a higher rate of residual polyp at follow-up colonoscopy compared to HSEMR. This data will need further validation with a larger sample size.

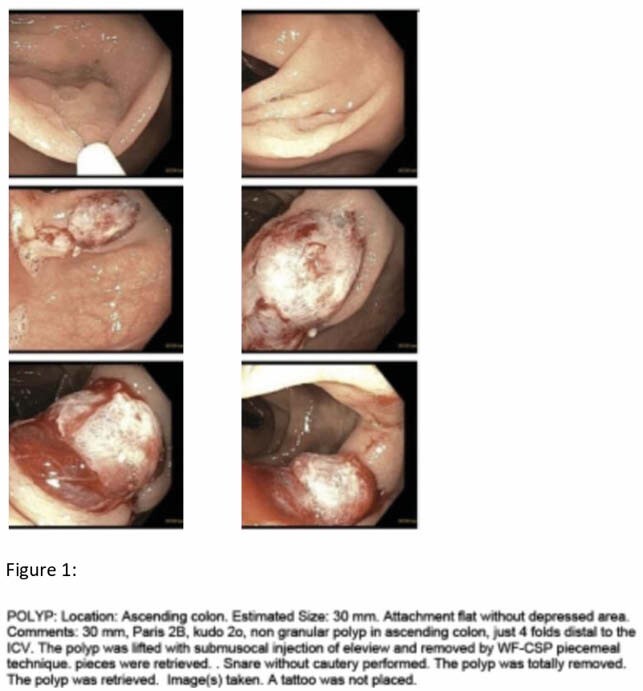

**Funding Agencies:**

None

